# Genomic Characterization of a Rare K30-ST198 Hypervirulent *Klebsiella pneumoniae* Clone with Distinctive Virulence Features

**DOI:** 10.3390/ijms26199601

**Published:** 2025-10-01

**Authors:** Domingo Fernández Vecilla, Jorge Rodríguez Grande, Nuria Fraile Valcárcel, Mary Paz Roche Matheus, Gotzon Iglesias Hidalgo, Cristina Aspichueta Vivanco, José Luis Díaz de Tuesta del Arco, Sergio García-Fernández, María Siller Ruiz, Zaira Moure, Daniela Vallejo Iriarte, Athanasia Varsaki, Jorge Calvo Montes, María Pía Roiz Mesones, María Carmen Fariñas, Alain A. Ocampo-Sosa

**Affiliations:** 1 Microbiology Service, University Hospital Marqués de Valdecilla, Av. Valdecilla s/n, 39008 Santander, Spain; sergio.garciaf@scsalud.es (S.G.-F.); maria.siller@scsalud.es (M.S.R.); zaira.moure@scsalud.es (Z.M.); jorge.calvo@scsalud.es (J.C.M.); mpia.roiz@scsalud.es (M.P.R.M.); 2 Marqués de Valdecilla Research Institute (IDIVAL), Av. Valdecilla s/n, 39008 Santander, Spain; jorge.rodriguez@idival.org (J.R.G.); nuria.fraile@idival.org (N.F.V.); daniela.vallejo@idival.org (D.V.I.); mcarmen.farinas@scsalud.es (M.C.F.); 3 CIBERINFEC, Health Institute Carlos III, Monforte de Lemos, 5, 28020 Madrid, Spain; 4 Basurto University Hospital, Montevideo Etorb., 18, Basurtu-Zorrotza, 48013 Bilbao, Spain; marypaz.rochematheus@osakidetza.eus (M.P.R.M.); cristina.aspichuetavivanco@osakidetza.eus (C.A.V.); joseluis.diazdetuestadelarco@osakidetza.eus (J.L.D.d.T.d.A.); 5 Cruces University Hospital, Cruces Plaza, 12, 48903 Barakaldo, Spain; gotzon.iglesiashidalgo@gmail.com; 6 Bioruces Health Research Institute, Cruces Plaza, 48903 Barakaldo, Spain; 7 Centro de Investigación y Formación Agrarias (CIFA), 39600 Muriedas, Spain; varsaki_a@cantabria.es; 8 Infectious Diseases Service, University Hospital Marqués de Valdecilla, Av. Valdecilla s/n, 39008 Santander, Spain

**Keywords:** hypervirulent *Klebsiella pneumoniae*, K30-ST198, ICEKp1, biofilm formation, *Galleria mellonella* infection model, virulence genes

## Abstract

Hypervirulent *Klebsiella pneumoniae* (hvKp) has emerged as a significant public health concern, yet rare sublineages remain poorly characterized. Here, we described a K30-ST198 hvKp sublineage identified in four isolates from two patients, including three sequential strains (K30B1, K30B2, K30B3) recovered over eight months from recurrent liver abscesses and one strain (K30-HUMV1) from a urinary tract infection. All isolates exhibited a yYpermucoviscous phenotype and resistance restricted to ampicillin and amoxicillin. Screening with the eazyplex hvKp assay detected *ybt* and *rmpA* in all strains, yielding a virulence score of 1. Biofilm production was strong in K30B1, K30B2, moderate in K30-HUMV1, but weak in K30B3. In the *Galleria mellonella* infection model, K30B1 showed higher virulence than the other isolates. Whole-genome sequencing identified the ICEKp1 carrying hypervirulence-associated genes (*ybt*, *pagO*, *rmpAC*, *iroBCDN*) together with additional virulence factors (*fim*, *mrkD*, *uge*, *ureA*, *wabG*, *wcaJ*, *mliC*), while antibiotic resistance genes were limited to *fosA* and *bla*_SHV-77_. Protein structures and their functional domains were predicted using AlphaFold v3.0.1 and ColabFold v1.5.5, based on pLDDT scores, providing further insights into gene functionality. This work represents one of the first detailed characterizations of K30-ST198 hvKp, underscoring the need for integrated genomic, phenotypic, and structural approaches in hvKp surveillance.

## 1. Introduction

Hypervirulent *Klebsiella pneumoniae* (hvKp) has emerged as a clinically significant pathotype that evolved from classical *K. pneumoniae* (cKp) and is responsible for severe community-acquired infections in otherwise healthy hosts. The syndrome was first described in Taiwan in the 1980s, when hvKp was recognized as the leading cause of cryptogenic pyogenic liver abscesses with a tendency for metastatic dissemination to the eye, central nervous system, and lungs [1,2]. Since then, hvKp has become an important pathogen worldwide, and its increasing prevalence outside Asia represents a major public health concern [2].

Unlike cKp, hvKp is characterized by distinctive virulence traits. The hallmark phenotype of hypermucoviscosity, typically identified by the string test, is largely mediated by the regulators of capsule synthesis genes *rmpA* and *rmpA2*, and occasionally by *magA* (traditionally associated with K1 serotype) [3]. Another major feature is the overproduction of siderophores, which allows hvKp to thrive in iron-restricted host environments. In particular, aerobactin (*iucABCD-iutA*) and salmochelin (*iroBCDN*) are strongly enriched in hvKp and are considered critical determinants of hypervirulence, while enterobactin and yersiniabactin are more widely distributed among both cKp and hvKp lineages [4,5]. Additional markers, such as the metabolite transporter *peg-344* [6], colibactin (*clbA-Q*) [7], and the allantoin metabolism operon (*allABCDRS*) [8], have been associated with increased pathogenic potential. However, the most recent and accurate definition for predicting hvKp strains includes the presence of all five virulence-associated genes: *iucA*, *iroB*, *peg-344*, *rmpA* and *rmpA2* [9], providing a pragmatic alternative to murine models.

Molecular epidemiology studies have shown that hvKp is predominantly associated with K1 and K2 capsular serotypes, with clonal groups CG23, CG65, and CG86 frequently implicated in invasive disease [10,11]. However, capsular types K5, K20, K54, and K57 have also been reported in hvKp strains, although less frequently [1]. The ability of hvKp to produce a thick capsule confers resistance to complement-mediated killing and phagocytosis, enhancing survival in the bloodstream [12]. Moreover, hvKp frequently carries plasmid-borne virulence loci, and concerningly, recent reports highlight the convergence of hypervirulence and multidrug resistance (MDR), including carbapenemase-producing hvKp strains [13,14]. This convergence poses a serious therapeutic challenge, as treatment options for such strains are extremely limited [15].

Although hvKp was initially considered an Asian problem, cases have been increasingly reported in Europe, North America, and South America [16,17,18]. Liver abscess remains the prototypical clinical manifestation, but hvKp infections now encompass a broad-spectrum including pneumonia, urinary tract infection, meningitis, osteomyelitis, and soft tissue infections [3]. The emergence of hvKp in new geographical settings underscores the importance of genomic surveillance to track the spread of high-risk clones. In line with this, a recent genomic study from Colombia reported the first characterization of hvKp isolates in the region, including a strain identified as ST198 with a K30 capsular type carrying classical hypervirulence determinants [19].

In this study, we describe four hvKp isolates belonging to the rare K30-ST198 sublineage, including one from a patient with a urinary tract infection and three from recurrent bloodstream infections in the same patient over an eight-month period. We characterized the capsular serotype, multilocus sequence type (MLST), and hypervirulence determinants using whole-genome sequencing (WGS). In addition, we evaluated biofilm production and virulence using the *Galleria mellonella* infection model. To our knowledge, this is the first report of hvKp K30-ST198 strains in our country.

## 2. Results

### 2.1. Clinical Data and Antimicrobial Susceptibility

Two patients were included in this study, each yielding *K. pneumoniae* isolates. The first case was a 72-year-old woman with no known comorbidities who presented with symptoms of uncomplicated urinary tract infection. A *K. pneumoniae* isolate (K30-HUMV1) was recovered from urine culture. The strain exhibited a hypermucoviscous phenotype and susceptibility to all tested antibiotics except ampicillin and amoxicillin. The patient was treated with oral cefuroxime (500 mg/12 h for 5 days) and experienced favorable clinical evolution without complications.

**Figure 1:**
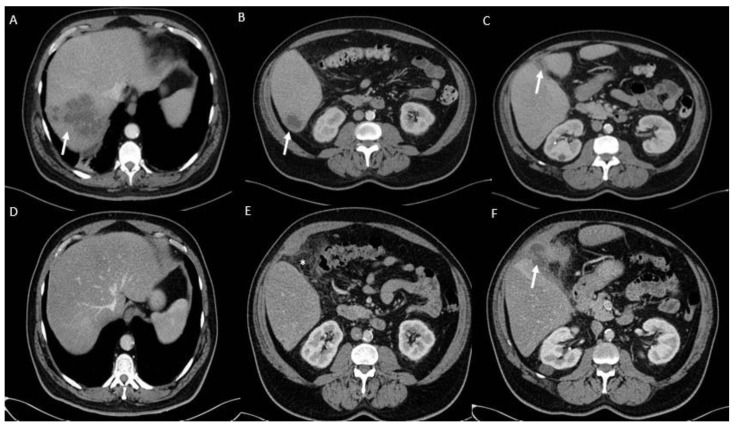
**Abdominopelvic computed tomography scan after contrast in the venous phase**. Several liver abscesses are seen as polylobulated hypodense lesions with peripheral enhancement in segments 7 (**A**) and segment 6 (**B**), while there is no abscess around the gallbladder bed (**C**). Resolution of the hepatic abscesses is seen in segments 7 (**D**) and segment 6 (**E**). However, a new abscess has developed in the gallbladder bed (arrow in (**F**)) with significant inflammatory changes in the surrounding mesocolonic fat, which were not present previously.

The second case involved a 57-year-old man with chronic liver disease and obesity. In January 2022, he was admitted with acute cholecystitis and underwent cholecystectomy. A *K. pneumoniae* isolate (K30B1) was recovered from bile. The patient responded well to intravenous piperacillin/tazobactam (4 g/0.5 g daily). Two months later (March 2022), he returned with fever and abdominal pain. An abdominopelvic computed tomography (CT) scan revealed multiple septated liver abscesses (Figure 1A–C), from which a second isolate (K30B2) with an identical phenotype and susceptibility profile was recovered. The patient was treated with drainage and a four-week course of IV antibiotics, achieving good outcome. A third episode occurred in September 2022, when a new abscess near the gallbladder bed was identified (Figure 1D–F). The isolate (K30B3) was again phenotypically identical to the previous strains. He was treated with drainage, IV antibiotics, and a short course of oral ciprofloxacin, resulting in complete clinical and radiological recovery.

Similarly to the isolate from the first case, these three strains were resistant only to ampicillin and amoxicillin, while remaining susceptible to all other tested antibiotics. Both patients were followed for several months after the resolution of the infection, and no recurrence was observed during the follow-up period.

### 2.2. String Test Results

The hypermucoviscous phenotype was confirmed in all four isolates by the string test [20]. Each strain produced a viscous string measuring > 5 mm when stretched with an inoculation loop from a colony grown on blood agar. The positive string test was consistently observed across three independent replicates, confirming the stable hypermucoviscous phenotype of K30B1, K30B2, K30B3, and K30-HUMV1.

### 2.3. Screening by eazyplex^R^ hvKp Assay

The eazyplex^®^ hvKp assay was performed on the four *K. pneumoniae* isolates (K30B1, K30B2, K30B3, and K30-HUMV1). The test consistently detected the siderophore gene *ybt* and the regulator of mucoid phenotype *rmpA* in all strains. No additional hvKp-associated loci, such as *iuc*, *iro*, or *peg-344*, were identified by this assay. According to the Kleborate classification scheme [21], the presence of *ybt* and *rmpA* corresponded to a virulence score of 1.

### 2.4. Determination of Biofilm Production

The ability of hvKp isolates to produce biofilm was assessed using a modified crystal violet staining assay [22]. Mean OD_600_ values ranged from 0.098 ± 0.050 in K30B2 to 1.210 ± 0.312 in K30B3. One-way ANOVA revealed significant differences in biofilm production among isolates (*F* = 24.98, *p* = 0.0002, *R*^2^ = 0.9036).

**Figure 2:**
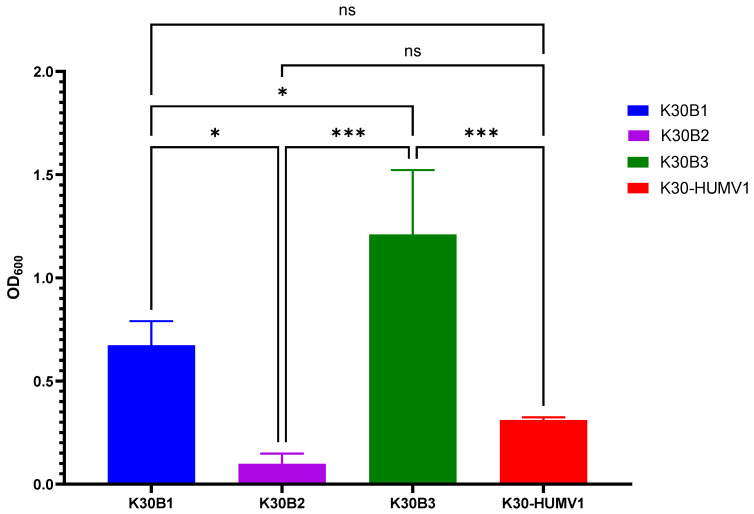
Quantification of biofilm formation by K30-ST198 hvKp isolates. Biofilm production was evaluated using a modified crystal violet (CV) staining method [21]. Bars represent the mean OD_600_ ± standard deviation from three independent experiments. Statistical significance was determined using one-way ANOVA followed by Tukey’s post hoc test. ns, not significant, * *p*-value < 0.05, *** *p*-value < 0.001.

Post hoc Tukey’s multiple comparisons test demonstrated that K30B3 exhibited significantly higher biofilm production compared with K30B1 (*p* = 0.0190), K30B2 (*p* = 0.0002), and K30-HUMV1 (*p* = 0.0008) (Figure 2). K30B1 produced significantly more biofilm than K30B2 (*p* = 0.0131), whereas no significant difference was observed between K30B1 and K30-HUMV1 (*p* = 0.1122). Similarly, K30B2 and K30-HUMV1 did not differ significantly (*p* = 0.4549).

Based on the classification criteria used (non-producer: OD ≤ 0.05, weak producer: OD > 0.05–0.1, moderate producer: OD > 0.1–0.3, and strong producer: OD > 0.3) [22], K30B3 (1.210 ± 0.312) and K30B1 (0.673 ± 0.117) were classified as strong biofilm producers, K30-HUMV1 (0.312 ± 0.012) as a moderate producer, and K30B2 (0.098 ± 0.050) as a weak producer. Importantly, these findings indicate a heterogeneous biofilm-producing capacity among hvKp isolates, which may contribute differently to their persistence and pathogenicity in host environment.

### 2.5. Galleria Mellonella Larvae Infection Model

**Figure 3:**
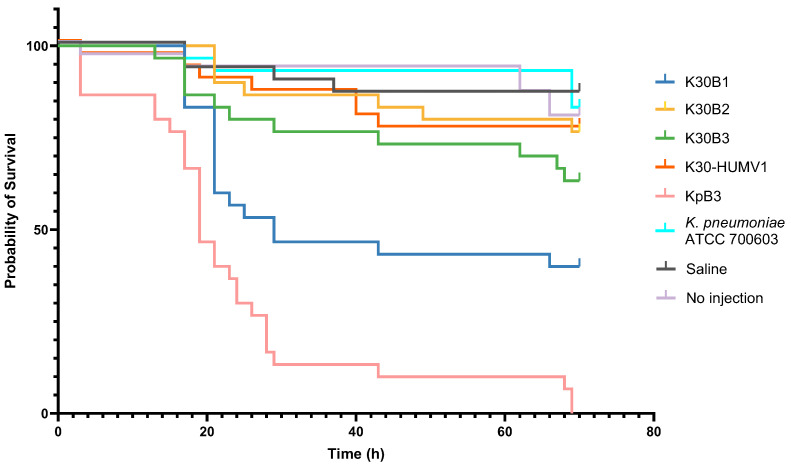
**Survival curves of *G. mellonella* larvae infected with hvKp isolates.** Larvae (n = 10 per group, tested in triplicate) were infected with 1 × 10^5^ CFU of the indicated *K. pneumoniae* isolates or with controls (saline or no injection). Survival was monitored over 72 h at 37 °C. The control strain KpB3 exhibited the highest virulence, causing progressive mortality and reaching 100% lethality at 69 h, followed by K30B1, which significantly reduced larval survival compared to low-virulence isolates. In contrast, K30B2, K30B3, and K30-HUMV1 demonstrated attenuated virulence, with survival rates exceeding 60% at the end of the experiment. The reference strain *K. pneumoniae* ATCC 700603 caused minimal mortality, comparable to saline-injected and untreated controls. Statistical significance was determined using the log-rank (Mantel–Cox) test: KpB3 vs. all other isolates, *p* < 0.001; K30B1 vs. K30B2 and K30B3, *p* = 0.01 and *p* = 0.03, respectively; no significant differences were observed among K30B2, K30B3, and K30-HUMV1 (*p* > 0.05).

The virulence potential of hvKp isolates was assessed in *G. mellonella* larvae by monitoring survival over 72 h post-infection (Figure 3). Clear differences in pathogenicity were observed among the clinical isolates K30B1, K30B2, K30B3, and K30-HUMV1.

K30B1 displayed the highest virulence among this sublineage. Larval mortality began within the first 24 h post-infection, with survival dropping below 50% by 43 h. By the end of the observation period, only ~40% of larvae remained alive. Comparative analyses confirmed that K30B1 was significantly more virulent than K30B2 (*p* = 0.01) and K30B3 (*p* = 0.03), though less virulent than the clinical control strain KpB3 (*p* = 0.002).

In contrast, K30B2, K30B3, and K30-HUMV1 exhibited markedly attenuated virulence. For these isolates, survival curves remained relatively stable during the first 48 h, with >70% of larvae alive, and more than 60% survival maintained at 72 h. Statistical comparisons indicated no significant differences among these three isolates (*p* > 0.05). This suggests that despite sharing a common genetic background, K30B2, K30B3, and K30-HUMV1 caused only limited lethality in vivo.

As expected, the hypervirulent control strain KpB3 induced rapid mortality, with a 50% lethal time of approximately 20 h post-infection and complete larval death by 69 h. By contrast, no significant mortality was observed in larvae infected with the low-virulence reference strain *Klebsiella quasipneumoniae* subsp. *similipneumoniae* ATCC 700603 [23] (hereafter referred to as *K. pneumoniae* ATCC 700603), or in saline-injected and untreated controls (*p* > 0.05).

### 2.6. Genetic Characterization by Whole-Genome Sequencing (WGS)

**Figure 4:**
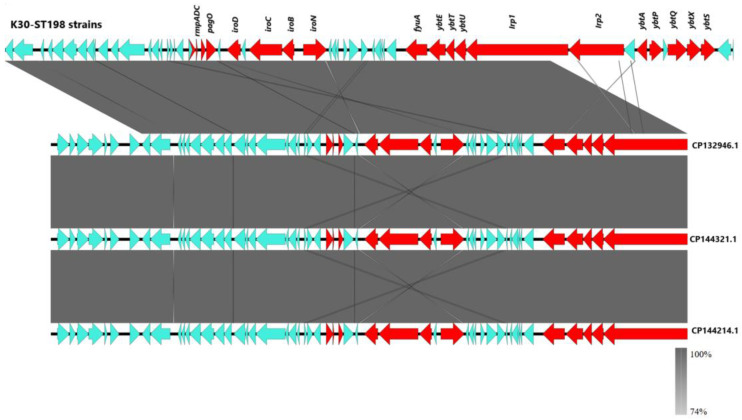
**Comparative genomic map of the 64-kbp ICEKp1 element in K30-ST198 hypervirulent *K. pneumoniae* isolates.** The schematic representation was generated with Easyfig [22] and compared with related sequences from GenBank (CP132946.1, CP144321.1, and CP144214.1). A single ICEKp1 sequence from one representative K30-ST198 isolate was used as all four study isolates displayed >99.5% identity and 100% coverage. The GenBank reference sequences also showed >99% identity and complete coverage. Genes involved in virulence, including *pagO*, *rmpADC*, the salmochelin (*iroBCDN*) operon and the *yersiniabactin* (*ybt*) operon are highlighted in red, while the remaining genes are in blue. green.

WGS confirmed that all four isolates belonged to the K30 capsular serotype and sequence type ST198 (K30-ST198), indicating they were part of the same sublineage. A 64-kb integrative and conjugative element (ICEKp1) was identified in all strains. A schematic representation of ICEKp1 was constructed using Easyfig [24], showing that all four isolates carried nearly identical elements (>99.5% sequence identity and 100% coverage). For simplicity, a single representative ICEKp1 sequence was used, as no substantial differences were observed among isolates. Comparative analysis with publicly available ICEKp1 sequences (GenBank accessions CP132946.1, CP144214.1, and CP144321.1) revealed >99% similarity and complete coverage, underscoring the high conservation of this element (Figure 4).

The ICEKp1 element carried key hypervirulence-associated loci, including the siderophore yersiniabactin (*ybt*), the regulators *rmpADC*, the *pagO* and *iroBCDN* operons, as well as genes encoding a type IV secretion system and a gene encoding a SAM-dependent methyltransferase. In addition, the four genomes contained further virulence-associated genes, such as *mrkB*, *mrkD*, *fimA*, *fimD*, *uge*, *ureA*, *wabG*, *wcaJ*, and *mliC*, all of which have been implicated in colonization, adhesion, and capsule biosynthesis [25,26,27,28,29].

Comparative variant analysis using Snippy v4.6.0 [30], revealed several mutations within classical hypervirulence regulators. Premature stop codons or frameshift mutations were identified in both *rmpA*, *rmpC and rmpD* across isolates, suggesting functional disruptions. The *pagO* locus remained intact in K30B1 and K30B2, whereas in K30B3 and K30-HUMV1, mutations produced premature stop codons consistent with pseudogenization (ψ*pagO*). The siderophore-associated gene *iroC* displayed extensive amino acid substitutions in all isolates; however, frameshift mutations resulted in pseudogenization in K30B1 and K30-HUMV1 (ψ*iroC*).

Interestingly, mutational analysis of biofilm-associated loci (*mrkB*, *mrkD*, *fimA*, and *fimD*) revealed notable differences among the four isolates. Within the type 3 fimbrial cluster, *mrkB* was intact in K30B1 and K30B2, but disrupted in K30B3 and K30-HUMV1 (ψ*mrkB*). The *mrkD* gene was conserved in three isolates but pseudogenized in K30B3 (ψ*mrkD*). Similarly, *fimA* was truncated in K30B1 and K30B3 (ψ*fimA*), while K30B2 and K30-HUMV1 had missense variants. Finally, *fimD* was intact in K30B1 and K30B2 (harboring non-conservative and semi-conservative substitutions), but classified as a pseudogene in K30B2 and K30-HUMV1 due to premature stop codons (ψ*fimD*).

Collectively, these findings demonstrate that K30-ST198 isolates harbor widespread disruptions in biofilm- and virulence-related loci, with recurrent pseudogenization affecting *rmpA*, *rmpC*, *rmpD*, *pagO*, *iroC*, *mrkB*, *mrkD*, *fimA*, and *fimD*, suggesting a convergent pattern of gene inactivation within this lineage. A detailed summary of mutational profiles is provided in Supplementary Material, Table S1.

Analysis of the resistome and mobilome revealed that no plasmids were present in any of the four K30 isolates. Furthermore, the only antimicrobial resistance genes detected were *fosA* and *bla*_SHV-77_.

### 2.7. Protein Structure Predictions and Functional Analysis

To further evaluate the functional consequences of observed mutations in hypervirulence- and biofilm-associated loci, protein structure predictions were generated using AlphaFold v3.0.1 [31] and ColabFold v1.5.5 [32]. Model reliability was assessed through pLDDT confidence scores, and structural features were examined for potential disruptions of functional domains.

Disruptions in the transcriptional regulator RmpA were commonly observed among the K30 isolates. The wild-type protein displays a compact architecture with multiple α-helices and β-strands modeled at high confidence, consistent with its regulatory role (Supplementary Figure S1). In isolate K30B1, *rmpA*, carried a ΔG_285_ deletion introducing a premature stop codon (TAA) at position 340, resulting in a truncated protein of 113 amino acids (aas) compared to the wild-type sequence (211 aas). Structural modeling confirmed the loss of critical domains required for transcriptional activation of capsule synthesis (Supplementary Material, Table S1 and Figure S1). K30B2 and K30B3 carried a ΔT_70_ mutation in *rmpA* generating an early stop codon at residue 79, while in K30-HUMV1, an Ins(C)_45_ insertion resulted in a stop codon at position 55, yielding a severely truncated product. Together, these mutations strongly suggest pseudogenization of *rmpA* despite persistence of the hypermucoviscous phenotype (Supplementary Material, Table S1).

All isolates also carried disruptions in *rmpC*, where frameshift mutations or substitutions introduced premature stop codons. These truncations, occurring between nucleotide positions 46 and 304, depending on the strain, resulted in loss of structural integrity and abolished most predicted functional domains (Supplementary Material, Table S1).

Sequence analysis of the *rmpD* gene revealed distinct genetic alterations among the K30-ST198 isolates. In isolate K30B3, insertion of the nucleotides TC at position 105 shifted the canonical stop codon TAG from position 123 to position 139 (TGA), extending the open reading frame by 16 nucleotides (Supplementary Material, Table S1). This mutation resulted in the production of a longer RmpD protein of 46 amino acids, compared with the 40-amino-acid wild-type protein. To evaluate whether this extension altered the predicted structure, protein models were generated using AlphaFold v3.0.1 (Supplementary Material, Figure S2). Both the wild-type and K30B3 variant proteins were predicted to form a continuous α-helical structure, with the six additional residues in the variant incorporated at the N-terminus without disrupting the overall fold. pLDDT confidence scores indicated very high reliability across most residues, with only the terminal ends and the added residues showing lower scores, consistent with structural flexibility. By contrast, a deletion of a guanine at position 62 in the *rmpD* sequence of isolates K30B1, K30B2, and K30-HUMV1 introduced a premature stop codon TGA at position 85. This truncation event supports the classification of *rmpD* in these isolates as a pseudogene (ψ*rmpD*) (Supplementary Material, Table S1).

In *pagO*, K30B1 and K30B3 carried intact wild-type alleles. By contrast, *pagO* in K30B2 carried a ΔA_169_ mutation that introduced a frameshift and generated a premature stop codon at position 247, whereas in K30-HUMV1 a G→T transversion at nucleotide position 85 produced an immediate stop codon (TAA). In both cases, these mutations resulted in pseudogenization of *pagO* (ψ*pagO*) (Supplementary Material. Table S1).

Predicted structural models of IroC revealed marked differences between the wild-type and variant proteins (Supplementary Material, Figure S3). The wild-type IroC protein consisted of 1218 amino acids, whereas the variant from isolate K30B1 was truncated to 978 amino acids, due to a ΔC_2856_ deletion leading to a premature stop codon at nucleotide position 2935, resulting in loss of the C-terminal region (Supplementary Material, Figure S2), while in K30-HUMV1, an Ins(CA)_65_ insertion generated a premature stop codon at position 100, consistent with pseudogenization (ψ*iroC*). The variant IroC protein from K30B2 and K30B3 exhibited multiple amino acid substitutions, which appear to slightly alter its structure but not its folding (Supplementary Material, Figure S2). These observations suggest that while the K30B1 mutation is likely to impair IroC function, the variants from K30B2 and K30B3 may largely preserve structural integrity.

Biofilm-associated proteins displayed heterogeneous alterations. The fimbrial chaperone MrkB was predicted to adopt a β-barrel domain with a short α-helical C-terminal tail in the wild type (Supplementary Figure S4). Structural confidence was high across the core β-barrel, while the C-terminal region displayed lower pLDDT values. Multiple alterations were identified in different isolates. In K30B1 and K30B3, two amino acid substitutions (D119N, I156T) preserved the global β-barrel architecture but disrupted the C-terminal α-helix, which appeared unstructured in the predicted models. In K30B2, a ΔT_23_ deletion introduced a premature stop codon at position 109, resulting in a truncated protein and pseudogenization of *mrkB* (ψ*mrkB*). Similarly, in K30-HUMV1, consecutive deletions at nucleotides 100 and 101 (ΔG_100_, ΔA_101_) generated a premature stop codon at position 127, also consistent with pseudogenization of *mrkB* and loss of functional domains (Supplementary Table S1).

The fimbrial adhesin MrkD also showed several variations (Supplementary Figure S5). MrkD proteins from isolates K30B1 and K30B3 only differed from the WT protein by one amino acid (E141Q), and K30-HUMV1 by three amino acids (E141Q, N195D, M196L); these variants retained the global β-sheet and C-terminal α-helix. In contrast, K30B2 harbored a ΔA_584_ mutation, introducing an early stop codon at residue 586, leading to a mutated MrkD protein of 195 amino acids in contrast to the complete protein of 331 amino acids. These modifications appear to affect the functional Domain 2 (residues 186–329), as defined by the CATH homologous superfamily 2.60.40.1090, based on high-confidence AlphaFold predictions (average pLDDT score: 97.61).

The wild-type major fimbrial subunit FimA (182 aa) was predicted to form a compact β-sheet N-terminal domain and an extended, unstructured C-terminal region (Supplementary Figure S6). In K30B3 and K30-HUMV1 strains, the FimA variants exhibited an identical structure without evident conformational differences despite presenting three amino acid substitutions (A19T, S74A, T151P). However, in K30B1 and K30B2 frameshift mutations produced truncated peptides of 34 and 56 amino acids, respectively, abolishing structural integrity (Supplementary Material, Table S1).

The usher protein FimD (828 aa) displayed a large β-barrel–rich structure with multiple domains and high-confidence scores in the wild type (Supplementary Figure S7). FimD variants from isolates K30B1 and K30B3 presented two substitutions (E48K, D436N) that did not alter structural organization. Conversely, truncations were observed in K30B2 (577 aa, ΔA_1451_/Δ_A1487_) and K30-HUMV1 (329 aa, ΔA_959_/ΔT_960_), both of which also carried multiple substitutions. These truncated models exhibited loss of distal domains and destabilization of the β-barrel architecture, consistent with loss of usher activity.

Overall, the integration of WGS data and structural predictions highlighted a complex relationship between genotype and phenotype in K30-ST198 isolates, with evidence of pseudogenization in several hypervirulence loci but retention of hypermucoviscosity, variable biofilm formation, and moderate-to-high virulence in vivo.

## 3. Discussion

This article describes the genomic and phenotypic characteristics of a rare K30-ST198 hvKp clone isolated from two patients, including three consecutive strains from a relapsed infection of cholecystitis and liver abscesses, and one strain from a urinary tract infection. Clinically, all isolates exhibited a hypermucoviscous phenotype and antimicrobial resistance limited to ampicillin and amoxicillin, a pattern consistent with classical hvKp strains that typically remain susceptible to most antimicrobials. Despite this common background, the isolates displayed striking heterogeneity in their biofilm-forming capacity and virulence potential, underscoring the need to complement genomic data with functional assays when evaluating hvKp strains.

Biofilm formation assays further illustrate this variability. Notably, K30B3 produced the highest biofilm biomass, far exceeding the values observed in the other strains and clearly classifying it as a strong biofilm producer. This suggests that K30B3 may possess enhanced biofilm-associated traits, such as increased extracellular polymeric substance production or the expression of biofilm-promoting surface adhesins, which could confer a survival advantage in hostile environments or during host colonization. K30B1 also displayed strong biofilm production, albeit at a lower level than K30B3, indicating that while both strains share the capacity for robust biofilm development, quantitative differences exist that may reflect strain-specific regulatory or metabolic factors. K30-HUMV1, positioned just above the strong producer threshold, showed a comparatively modest biofilm yield, suggesting that under the tested conditions it maintains a moderate ability to establish biofilms, which could be enhanced or suppressed depending on environmental signals. In contrast, K30B2 exhibited only weak biofilm formation. This could be due to reduced expression of biofilm-related genes, impaired production of extracellular matrix components, or a growth phenotype that disfavors surface attachment. Fimbrial genes exhibited diverse mutational outcomes, with potential consequences for adhesion and biofilm formation. Importantly, the observed biofilm phenotypes corresponded well to predicted genotypes: K30B2 exhibited weak biofilm production due to the presence of truncations in *mrkB*, *mrkD*, *fimA*, and *fimD*, whereas K30B3 produced the strongest biofilms by carrying only minor substitutions. However, biofilm development in *K. pneumoniae* should be considered multifactorial, influenced not only by structural gene integrity but also by compensatory pathways and regulatory networks. Such intra-species variability has been documented previously [33,34], reinforcing the complexity of biofilm biology. Clinically, strong biofilm producers such as K30B3 may pose a higher risk, as biofilms are known to enhance tolerance to antimicrobials and protect against host immune clearance [35].

In vivo testing using the *G. mellonella* model further demonstrated heterogeneity in virulence within the K30-ST198 sublineage. Among the clinical isolates, K30B1 exhibited the greatest pathogenic potential, causing rapid larval mortality within the first 24 h and reducing survival to below 50% by 43 h. By the end of the observation period, only ~40% of larvae remained alive. Although less virulent than the hypervirulent control strain KpB3, which achieved complete lethality by 69 h, K30B1 was significantly more virulent than K30B2 and K30B3 (*p* = 0.01 and *p* = 0.03, respectively). In contrast, K30B2, K30B3, and K30-HUMV1 induced only limited lethality, maintaining >70% survival at 48 h and >60% at 72 h, with no significant differences among them (*p* > 0.05). These findings suggest that, despite their shared genetic background, isolates within this sublineage differ substantially in their ability to cause disease in vivo.

The observed attenuation in virulence of K30B2, K30B3, and K30-HUMV1 may reflect disruptions in specific virulence-associated loci, regulatory differences, or compensatory mechanisms that limit pathogenicity. Conversely, the persistence of virulence in K30B1 underscores the possibility that even within lineages characterized by pseudogenization of canonical hvKp markers, certain isolates retain clinically relevant pathogenic potential. The lack of mortality in larvae challenged with the low-virulence reference strain *K. pneumoniae* ATCC 700603 or in negative controls confirms that lethality was attributable to infection rather than experimental artifacts, supporting the validity of the *Galleria* model for detecting strain-dependent differences in pathogenicity. To further explore the genetic basis of these phenotypic differences, structural modeling of virulence-associated proteins was performed. The predicted models of IroC revealed that the consequences of mutation differ markedly between isolates. While the truncation observed in K30B1, and especially the premature stop codon in K30-HUMV1, are consistent with pseudogenization and probable loss of siderophore transport, the variants in K30B2 and K30B3 largely retained structural integrity, suggesting that function may be preserved. Such heterogeneity echoes findings in other hvKp lineages, where partial disruption of iron acquisition systems does not necessarily abolish virulence but may instead modulate fitness depending on the host niche.

Although all isolates were assigned a Kleborate score of only 1, WGS revealed the presence of multiple virulence determinants within ICEKp1, including *pagO*, *rmpADC*, *iroBCDN*, and *ybt*, the latter being the only locus contributing to the Kleborate score, as well as other virulence genes such as *fim*, *mliC*, and *wcaJ* [1,6,36]. Notably, the *rmpADC* operon exhibited multiple mutations across all K30-ST198 isolates, leading to pseudogenization of its components, especially *rmpA* and *rmpC*, and suggesting a loss of canonical functionality in this lineage. Within this context, detailed analysis of the *rmpD* gene, typically co-localized within the *rmpADC* operon and previously implicated as a critical driver of hypermucoviscosity [37], revealed two distinct genetic outcomes with potential phenotypic consequences. In isolates K30B1, K30B2, and K30-HUMV1, a guanine deletion at position 62 introduced a premature stop codon at position 85, generating a truncated gene product (ψ*rmpD*). The absence of a full-length RmpD in these isolates is consistent with disruption of its reported role in the regulation of hypermucoviscosity and may contribute to functional divergence within this clonal group. By contrast, isolate K30B3 harbored insertions that extended the open reading frame, producing a longer RmpD protein of 46 amino acids compared with the 40 amino acids in the wild type. Importantly, structural modeling predicted that this variant maintained the characteristic α-helical fold of wild-type RmpD, with the additional residues incorporated at the N-terminal region without destabilizing the protein. These findings indicate that the K30B3 variant retains structural conservation despite its altered length, suggesting that functional properties could be preserved. However, the lower model confidence at the extended N-terminus may reflect a region of increased flexibility that could influence interactions with other proteins or nucleic acids. The coexistence of ψ*rmpD* alleles and a structurally conserved elongated variant within the same sequence type illustrates the genetic plasticity of hvKp. Such heterogeneity likely contributes to phenotypic variability, including differences in virulence expression and biofilm formation. Indeed, K30B3 exhibited markedly higher biofilm production compared with isolates carrying ψ*rmpD*, suggesting that the presence of an intact or extended RmpD may provide a functional advantage in this context. Nonetheless, all isolates maintained hypermucoviscosity despite disruption of *rmpA* and *rmpC*, pointing toward compensatory regulatory pathways that preserve this hallmark virulence trait.

Our findings expand current knowledge of the rare K30-ST198 sublineage, which is characterized by a unique set of virulence factors that merit in-depth investigation. A recent study identified up to 21 novel virulence factors, including *pagO* and the *fecIRA* cluster, which appear to be associated with hvKp strains [38]. The authors investigated the association of these virulence genes with liver abscess formation by conducting a comparative analysis between PLA strains and non-PLA strains. The genes *pagO*, *wcaJ*, a gene encoding a putative SAM-dependent methyltransferase, and *mliC* (encoding a lysozyme inhibitor) were found in all strains studied. Notably, Tu YC et al. were the first to suggest that *pagO*, a DMT family transporter sharing 70% protein homology with *peg-344*, is required for PLA induction in hvKp [39]. Multiple studies have reported the presence of this gene in hvKp strains [38,39,40,41,42]. Ye M and Jin M et al. demonstrated that *pagO* was present in most PLA-causing strains, while being rarely found in isolates from other sites [38,40]. All of our strain carried the *pagO* together with other virulence determinants within an ICEKp1. Remarkably, the attenuated phenotype of truncated-*pagO* isolates (K30B2 and K30-HUMV1) compared to the wild-type carriers (K30B1 and K30B3) is consistent with previous reports supporting a role for *pagO* in virulence and liver abscess formation [38]. However, the relatively low virulence observed in K30B3, despite harboring an intact allele, indicates that *pagO* alone is not sufficient to confer hypervirulence. These findings suggest that while *pagO* contributes to the pathogenic potential of hvKp, its functional impact is likely modulated by strain-specific regulatory networks and metabolic background.

Although hvKp infections were previously restricted to specific capsular serotypes, hypervirulent strains are now more accurately defined by the presence of virulence genes such as *rmpA*, *rmpA2*, *iuc*, *iro* and *peg-344* [9]. The introduction of LAMP assays in laboratories allows rapid identification of target sequences with higher sensitivity than traditional PCR, while also being cost-effective [43]. The *eazyplex^®^ hvKp* kit, currently available as a research-use-only (RUO) assay, has the potential to enhance surveillance and enable early detection of these strains in under 30 min. Rödel J et al. used a combination of the LAMP-based *eazyplex^®^ Superbug CRE* and hvKp assays to simultaneously detect carbapenemases and virulence genes in 87 strains [44], successfully identifying 13 hvKp strains with a Kleborate score ≥ 3, six of which also carried carbapenemase genes. In our recent study [45], we evaluated the eazyplex^®^ hvKp assay using spiked blood cultures and demonstrated its potential for rapid hvKp detection directly from human clinical specimens. However, Russo TA et al. argue that the Kleborate score is not optimal for distinguishing hvKp from cKp for two main reasons [46]: (a) *ybt* and *clb* can be found in multiple Kp populations, and (b) aerobactin is weighted too heavily in the scoring system. For example, in our study, the strains scored only 1 point on the Kleborate scale despite carrying *rmpADC* and other virulence-associated genes such as *pagO* and *mliC*. These authors also recommend that a strain should not be classified as hypervirulent unless it possesses at least four of the five canonical virulence genes (*rmpA*, *rmpA2*, *iucA*, *iroB*, and *peg-344*) [47]. If this genetic profile is absent, they suggest confirming hypervirulence through an appropriate murine infection model rather than the *G. mellonella* model, arguing that the latter does not reliably distinguish hvKp from cKp [48]. Nevertheless, recent studies by Mai D and Li G et al. challenge this view [49,50], concluding that combining the *G. mellonella* model with genetic marker detection and quantitative siderophore determination can improve the sensitivity, specificity, and predictive value for hvKp identification. We would also like to emphasize the necessity of including WGS in these analyses, as simple identification by PCR or LAMP-based assays is not sufficient to determine whether the so-called five canonical hypervirulence-associated genes are intact or functional. Without this information, strains may be incorrectly classified as hypervirulent despite being potentially non-virulent in either *G. mellonella* or murine infection models.

These findings highlight the difficulty of defining hypervirulence based solely on a restricted set of genetic markers or infection models and emphasize the value of comprehensive genomic and phenotypic analyses. Alongside these considerations, the distribution of capsular serotypes also plays a critical role in pathogenic potential. The most common capsular serotypes associated with hvKp are K1, K2, K5, K20, K54, and K57, with K1 and K2 accounting for 70–75% of cases [1,51]. Infections caused by K30 serotype isolates are rarely reported. Attalla ET et al. described seven K30-ST383 colistin-resistant strains carrying *iuc* or *ybtA* virulence genes, though only one harbored *rmpA* [52]. Cubero M et al. reported 53 hvKp strains causing bacteremia from 2007 to 2013, of which two were K30 (one ST234 and one ST416) [53]. Lin YT et al. found that K1 and K2 accounted for 10% of *K. pneumoniae* strains from healthy individuals across several Asian countries, whereas only 1% carried the K30 serotype [54]. Hayashi W et al. identified K30-ST29 cKp strains resistant to colistin and tigecycline from wastewater influents in Japan [55]. Regarding ST198, Rada AM et al. first reported a K30-ST198 hvKp strain in Colombia causing meningitis, which carried ICEKp1 along with *rmpA*, *ybtA*, and *pagO* [19]. Notably, the Colombian strain harbored the same virulence determinants as those identified in our isolates, suggesting possible dissemination of this lineage across geographic regions and highlighting the importance of WGS-based epidemiological surveillance to monitor its spread.

## 4. Materials and Methods

### 4.1. Ethics Statement

This study was conducted in accordance with the ethical principles of the Declaration of Helsinki. The use and publication of clinical data were approved by the Research Ethics Committee of the Basurto University Hospital (code: 65.24 CEIHU), 04/02/2023. The requirement for written informed consent was waived by the committee due to the retrospective and observational nature of the study, in which the patients were no longer available at the time of the study and were difficult to locate.

### 4.2. Strain Isolation, Antimicrobial Susceptibility Testing (AST), and Clinical Data Collection

Four *K. pneumoniae* strains were analyzed in this study. One strain (K30-HUMV1) was isolated from a 72-year-old woman with no known comorbidities who presented with an uncomplicated urinary tract infection. The other three strains (K30B1, K30B2, K30B3) were isolated from a 57-year-old man with chronic liver disease and obesity, each corresponding to a separate relapse episode within an eight-month timeframe.

The strains were identified by using the Vitek MS matrix-assisted laser desorption/ionization time-of-flight (MALDI-TOF) mass spectrometry (BioMérieux, Marcy-l’Étoile, France). AST was performed using AST-N426 cards for *Enterobacterales* in the VITEK^R^ 2 system (BioMérieux, Marcy-l’Étoile, France), and results were interpreted according to the European Committee on Antimicrobial Susceptibility Testing (EUCAST) guidelines [56].

Relevant clinical data, including comorbidities, clinical presentation, treatment and disease evolution, were obtained from the medical records of both patients.

### 4.3. Identification of the Hypermucoviscous (HMV) Phenotype

The HMV phenotype was determined by the “string test,” in which colonies grown overnight at 37 °C were considered positive if they produced a viscous filament ≥5 mm when lifted with an inoculating loop [20].

### 4.4. Screening with the eazyplex^®^ hvKp Assay

Rapid virulence gene detection was performed using the Loop-mediated isothermal amplification (LAMP)-based “eazyplex^R^ hvKp” assay (Amplexdiagnostics GmbH, Gars, Germany), available exclusively for Research Use Only (RUO). This assay targets six virulence genes (*iucC*, *iroC*, *rmpA/rmpA2*, *ybt and clb*) directly from bacterial colonies. While detection follows the Kleborate score system [21], only *ybt*, *clb* and *iucC* are considered for score calculation.

### 4.5. Biofilm Production Assay

Biofilm formation was quantified using the crystal violet (CV) staining method with minor modifications from a previously described protocol [22]. Briefly, 100 μL of a 0.2 McFarland bacterial suspension were inoculated into 900 μL of Mueller-Hinton broth (MHB; BD Difco, Thermo Fisher Scientific Inc., Waltham, MA, USA) in each well of a 24-well plate (Nunc, Thermo Fisher Scientific Inc., Waltham, MA, USA), with three technical replicates per strain. Plates were incubated overnight at 37 °C. The supernatant containing planktonic cells was discarded, and wells were stained with 0.7% CV (Sigma-Aldrich, St. Louis, MO, USA) for 12 min.

Excess dye was removed by washing three times with sterile distilled water. Bound CV was solubilized in 33% (v/v) acetic acid (Sigma-Aldrich, St. Louis, MO, USA), and the optical density (OD) at 600 nm was measured using an Infinite^®^ microplate reader (Tecan Trading AG, Männedorf, Switzerland), with nine readings per well after shaking.

The procedure was repeated on three independent days. For the interpretation of the results, four cut-off points were established: non biofilm producer (OD ≤ 0.05), weak biofilm producer (OD > 0.05–0.1), moderate biofilm producer (OD > 0.1–0.3) and strong biofilm producer (OD > 0.3).

### 4.6. Virulence Assay in Galleria mellonella

Virulence of the isolates was evaluated using larvae of the greater wax moth (*G. mellonella*). Larvae (25 mm) were obtained from Harkito Reptile (Madrid, Spain) and maintained on woodchips at 10 °C in darkness until use. Bacterial isolates were cultured on Columbia blood agar (bioMérieux, Marcy-l’Étoile, France) and incubated overnight at 37 °C. Cell suspensions were prepared in 0.9% saline to a turbidity of 0.5 McFarland and adjusted to 1 × 10^5^ CFU/mL.

Groups of ten larvae per isolate were infected following a previously described protocol [22]. In brief, 10 μL of the adjusted bacterial suspension were injected into the last left proleg using a sterile insulin syringe (0.33 mm [29G] × 12.7 mm; 0.5 mL (Becton Dickinson, Franklin Lakes, NJ, USA). Two negative controls were included: larvae injected with saline and untreated larvae. The hypervirulent *K. pneumoniae* strain KpB3 (clinical isolate, unpublished) and *K. pneumoniae* ATCC 700603 [23] were employed as high-and low-virulence controls, respectively. Each strain was tested in triplicate.

Following infection, larvae were incubated at 37 °C and examined every 2–4 h over a 72-h period. Mortality was defined by the presence of melanisation and absence of response to tactile stimulation.

### 4.7. Whole-Genome Sequencing (WGS) and Bioinformatics Analysis

Genomic DNA was extracted using the Qiagen DNeasy^®^ Blood and Tissue Kit (Qiagen, Hilden, Germany) and sequenced on a MinION-Mk1B device (Nanopore Technologies, Oxford, UK) for 24 h in a single run, using the Rapid Barcoding Kit (SQK-RBK004) with an R9 flow cell (FLO-MIN106). Genome annotation was conducted using Bakta (https://github.com/oschwengers/bakta, accessed on 11 September 2024) [57]. Strains typing was performed using Kaptive 3.0 (https://github.com/klebgenomics/Kaptive, accessed on 19 June 2025) [58] and BIGSdb (https://bigsdb.pasteur.fr/klebsiella, accessed on 22 November 2024). The presence of integrative conjugative elements (ICE) was assessed using ICEfinder (https://tool2-mml.sjtu.edu.cn/ICEberg3/ICEfinder.php, accessed on 22 November 2024) [59]. Basic Local Alignment Search Tool (BLASTn, https://blast.ncbi.nlm.nih.gov/Blast.cgi?PROGRAM=blastn&BLAST_SPEC=GeoBlast&PAGE_TYPE=BlastSearch), accessed on 12 Novembre 2024) was used to identify relevant genes among the strains. ICEKp1 structure was visualized with Easyfig [22] and compared with reference sequences from GenBank. Single nucleotide polymorphisms (SNPs) and insertions/deletions (indels) were analyzed with Snippy v4.6.0 (https://github.com/tseemann/snippy, accessed on 18 February 2025) [29]. PlasmidFinder (https://github.com/genomicepidemiology/plasmidfinder, accessed on 3 November 2024) [60] and ResFinder (https://github.com/genomicepidemiology/resfinder, accessed on 3 November 2024) [61] tools available on the CGE website were employed to detect plasmids and acquired antimicrobial resistance genes, respectively. In addition, the Comprehensive Antibiotic Resistance Database (CARD, v3.2.8; https://card.mcmaster.ca/, accessed on 3 November 2024) was used to identify potential antimicrobial resistance determinants.

### 4.8. Protein Structure Prediction

Protein structure modeling and functional domain prediction were performed using AlphaFold v3.0.1 [31] and ColabFold v1.5.5 [32]. For each protein of interest, three-dimensional structures were generated and subsequently assessed for confidence using the predicted Local Distance Difference Test (pLDDT) scores. Functional domains were identified based on regions with high-confidence pLDDT values (>70), while low-confidence regions (<50) were interpreted with caution. Structural models were further inspected to evaluate potential domain organization and to support functional inference of virulence-associated genes identified through whole-genome sequencing.

### 4.9. Statistical Analyses

Biofilm production among the strains was compared using one-way ANOVA, followed by Tukey’s post hoc test for pairwise comparisons. Survival outcomes from the *G. mellonella* infection model were analysed with Kaplan–Meier survival curves, and group differences were assessed using the log-rank (Mantel–Cox) test with χ^2^ statistics. A *p*-value <0.05 was considered statistically significant. All statistical analyses were performed in GraphPad Prism v.10.5.0 (GraphPad Software, San Diego, CA, USA). Experiments were conducted in biological triplicate, and results are reported as means ± standard deviation.

## 5. Conclusions

This study provides the first detailed characterization of a rare K30-ST198 hypervirulent *K. pneumoniae* (hvKp) clone, isolated from two patients with distinct clinical presentations. All isolates displayed a hypermucoviscous phenotype and resistance limited to ampicillin and amoxicillin, consistent with classical hvKp susceptibility profiles. Despite this shared background, the strains exhibited striking heterogeneity in both biofilm formation and virulence, underscoring the need to complement genomic analyses with phenotypic assays to fully assess pathogenic potential.

Phenotypically, K30B2 emerged as the strongest biofilm producer, while K30B3 showed only weak formation, despite carrying fewer genetic disruptions in biofilm-associated loci. Similarly, virulence in the *G. mellonella* model varied markedly: K30B1 retained high pathogenic potential, approaching that of the hypervirulent control strain, whereas K30B2, K30B3, and K30-HUMV1 caused attenuated infections. These findings emphasize the multifactorial regulation of hvKp pathogenicity, where neither biofilm capacity nor the presence of canonical virulence determinants alone predict clinical risk.

Genomic analysis revealed that all isolates harbored a conserved ICEKp1 element encoding *pagO*, *rmpADC*, *iroBCDN*, and *ybt*, together with other virulence-associated genes. Analysis of the *rmpADC* operon in K30-ST198 isolates revealed extensive pseudogenization of *rmpA* and *rmpC*, alongside divergent evolutionary trajectories of *rmpD*. The coexistence of truncated ψ*rmpD* alleles and an elongated, structurally conserved variant underscores the genetic plasticity of this lineage and may underlie variability in virulence phenotypes such as biofilm formation. These observations highlight the potential for alternative regulatory pathways to sustain hypermucoviscosity in the absence of canonical *rmpADC* function.

Our findings expand current knowledge of the K30-ST198 sublineage and underscore the complexity of hvKp pathogenesis, where canonical markers alone are insufficient to predict virulence. They also emphasize the necessity of integrating genomic data, protein structural modeling, and functional assays, including in vivo infection models to accurately assess strain pathogenicity. Furthermore, the detection of virulence determinants such as *pagO* within ICEKp1 highlights the multifactorial nature of hypervirulence and its modulation by strain-specific genetic backgrounds. Given the global reports of K30-ST198, enhanced genomic surveillance is warranted to monitor dissemination of this lineage and its potential clinical impact.

## Data Availability

Data associated with this article are included in the Supplemental Materials: (Table S1, Figure S1). Whole-genome sequences of *K. pneumoniae* isolates K30B1, K30B2 and K30B3, K30-HUMV1 are available in the NCBI database under Bioproject/PRJNA1186724.

## Supplementary Materials

The following supporting information can be downloaded at https://www.mdpi.com/article/10.3390/ijms26199601/s1.
