# Supplementary material for: Genomic Characterization of a Rare K30-ST198 Hypervirulent Klebsiella pneumoniae Clone with Distinctive Virulence Features
Source: Int J Mol Sci. 2025 Oct 1;26(19):9601. doi: 10.3390/ijms26199601 (PMC12525063; doi:10.3390/ijms26199601)
Supplement: Supplementary file 1 [file ijms-26-09601-s001.zip › ijms-3884768-supplementary/Supplementary Figures S1-S7.pdf]

Supplementary Material. Figures S1-S7: Structural predictions of wild-type and variant proteins generated using AlphaFold and ColabFold

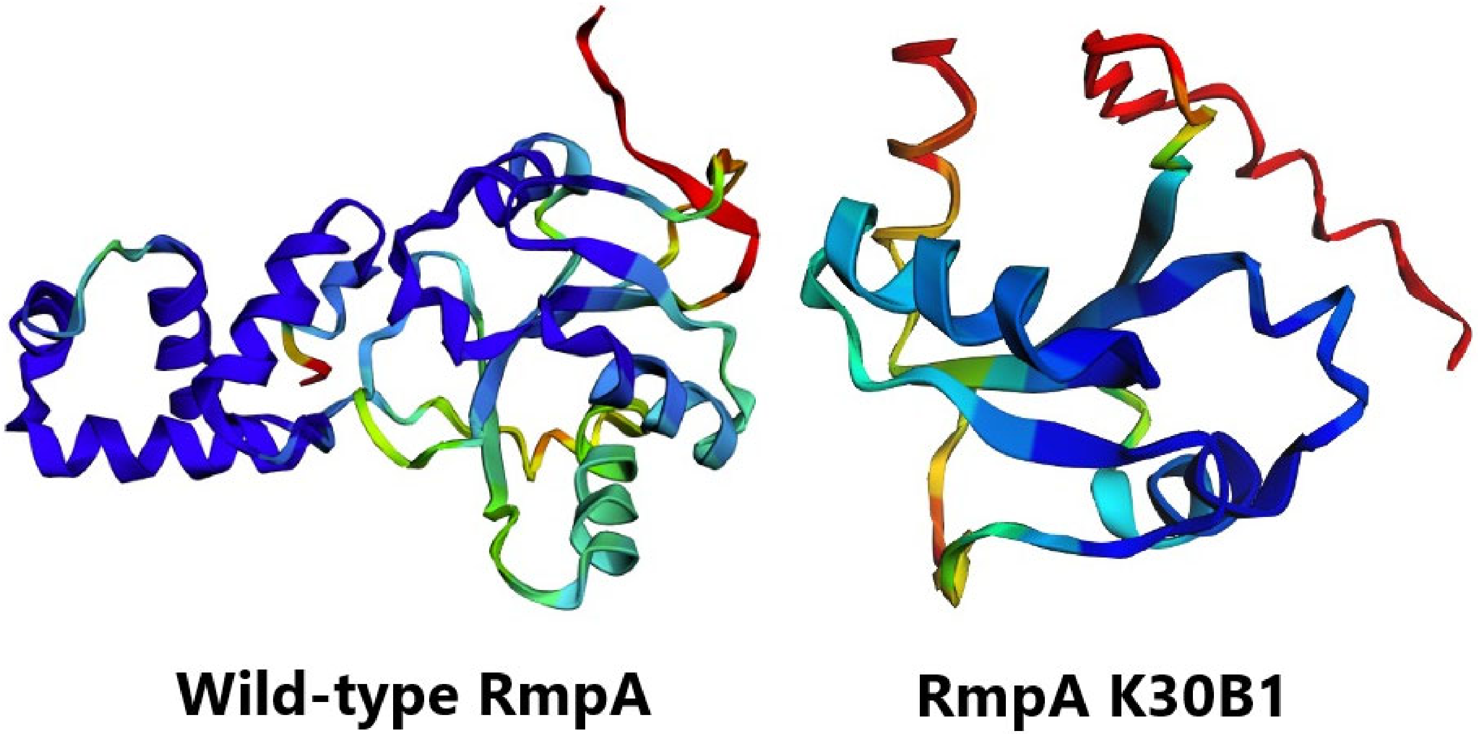

Supplementary Material. Figure S1. Predicted protein structures of wild-type and truncated RmpA. Structural models were generated using ColabFold v1.5.5 [31]. Since a truncated RmpA protein was not available in the AlphaFold database, the wild-type RmpA protein was modeled and compared with the truncated variant from isolate K30B1,

which carries a single-base deletion (ΔG281) in the *rmpA* gene leading to a premature stop codon at position 337 and a shortened protein of 112 amino acids. Models are colored by pLDDT confidence scores: blue (very high confidence), cyan–green (high to intermediate), yellow–orange (low), and red (very low confidence) in residue positioning.

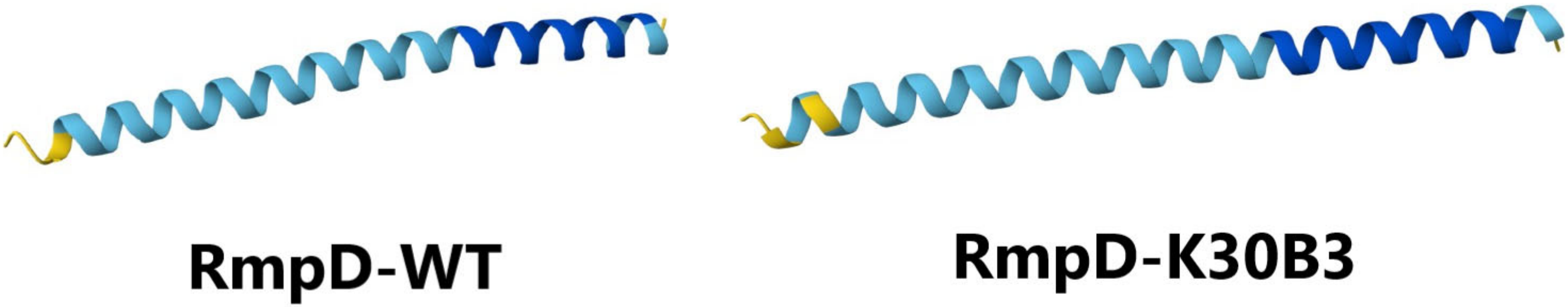

Supplementary Material. Figure S2. Predicted protein structures of wild-type and larger RmpD variant of isolate K30B3. Structural models were generated using AlphaFold v3.0.1 [30]. The wild-type RmpD protein (40 amino acids) was compared with the K30B3 variant (46 amino acids), which showed no structural alterations. Models are colored by pLDDT confidence scores: blue (very high confidence), cyan–green (high to intermediate), yellow–orange (low), and red (very low confidence) in residue positioning

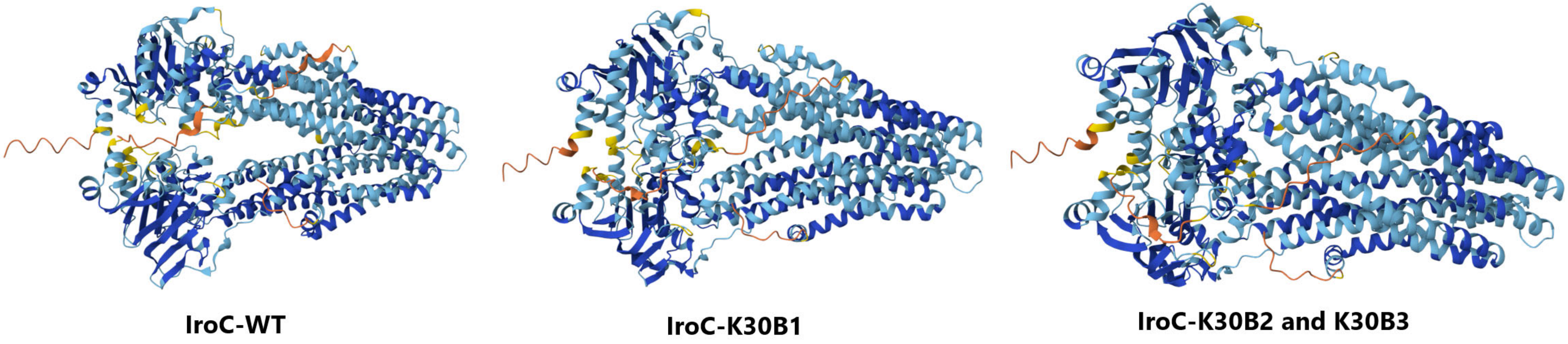

Supplementary Material. Figure S3. Predicted protein structures of wild-type and truncated IroC. Structural models were generated using AlphaFold v3.0.1 [30], the wild-type IroC protein of 1218 amino acids was compared with the IroC protein variant from isolate K30B1 and from the isolates K30B2 and K30B3. The variant IroC protein from K30B1 presented 978 amino acids because of a premature stop codon at position 2935 of the nucleotide sequence. In contrast, the variant IroC protein of K30B2 and K30B3 exhibited multiple amino acid substitutions, which appear to slightly alter its structure but not its folding. Models are colored by pLDDT confidence scores: blue (very high confidence), cyan–green (high to intermediate), yellow–orange (low), and red (very low confidence) in residue positioning.

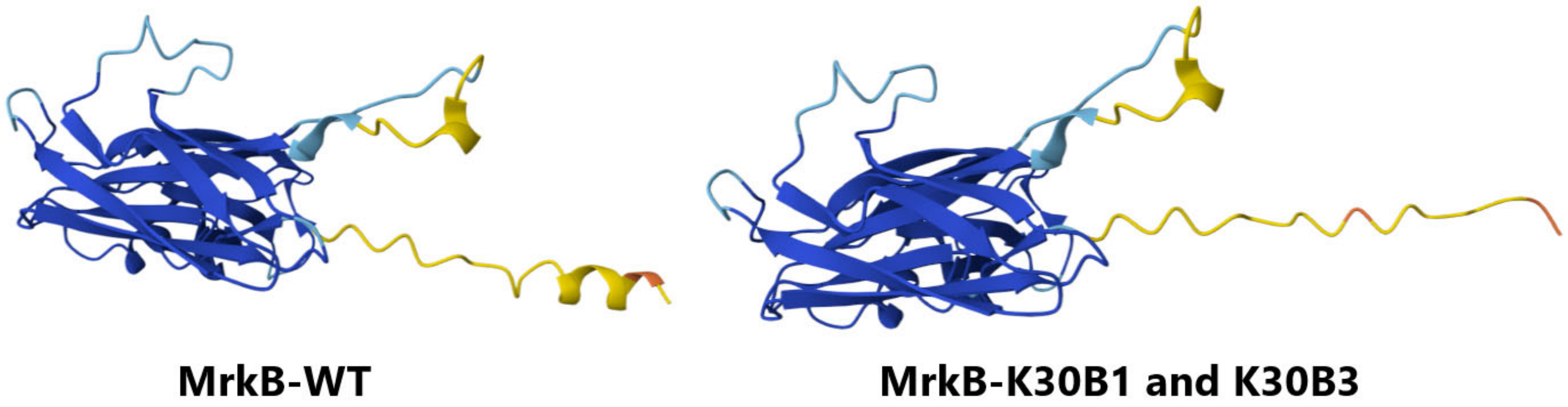

Supplementary Material. Figure S4. Predicted protein structures of wild-type and truncated MrkB. Structural models were generated using AlphaFold v3.0.1 [30]. The wild-type MrkB protein was modeled and compared with the mutated variants from isolates K30B1 and K30B3, which only presented two amino acid mutations (D119N, I156T). The C-terminal tail of MrkB (highlighted in yellow) shows a marked structural difference between the wild-type and the mutated variants. While in MrkB-WT it adopts a short α-helical conformation, in the K30B1 and K30B3 variants this region appears largely unstructured and extended, suggesting a loss of local secondary structure. Models are colored by pLDDT confidence scores: blue (very high confidence), cyan–green (high to intermediate), yellow–orange (low), and red (very low confidence) in residue positioning.

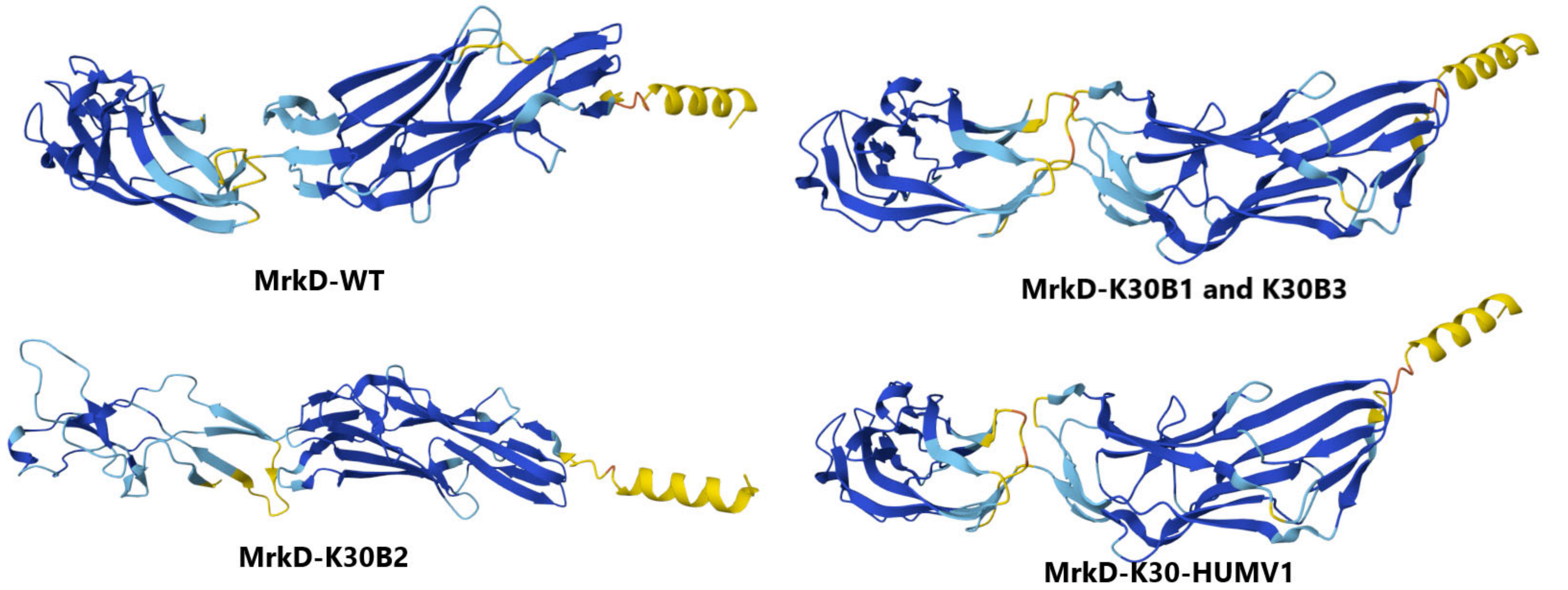

Supplementary Material. Figure S5. Predicted protein structures of wild-type and truncated MrkD. Structural models were generated using AlphaFold v3.0.1 [30]. The wild-type MrkD protein was modeled and compared with the truncated variant from isolate K30B2, which carries a single-base deletion (ΔA_{584}) in the *mrkD* gene leading to a premature stop codon at position 586 and a shortened protein of 195 amino acids. MrkD proteins of K30B1 and K30B2 exhibited only one amino acid mutation (E141Q), while K30-HUMV1 showed two additional mutations on the protein (E141Q, N195D, M196L). MrkD-K30B2 exhibits the most pronounced alteration, with a more open and less compact fold. Models are colored by pLDDT confidence scores: blue (very high confidence), cyan–green (high to intermediate), yellow–orange (low), and red (very low confidence) in residue positioning.

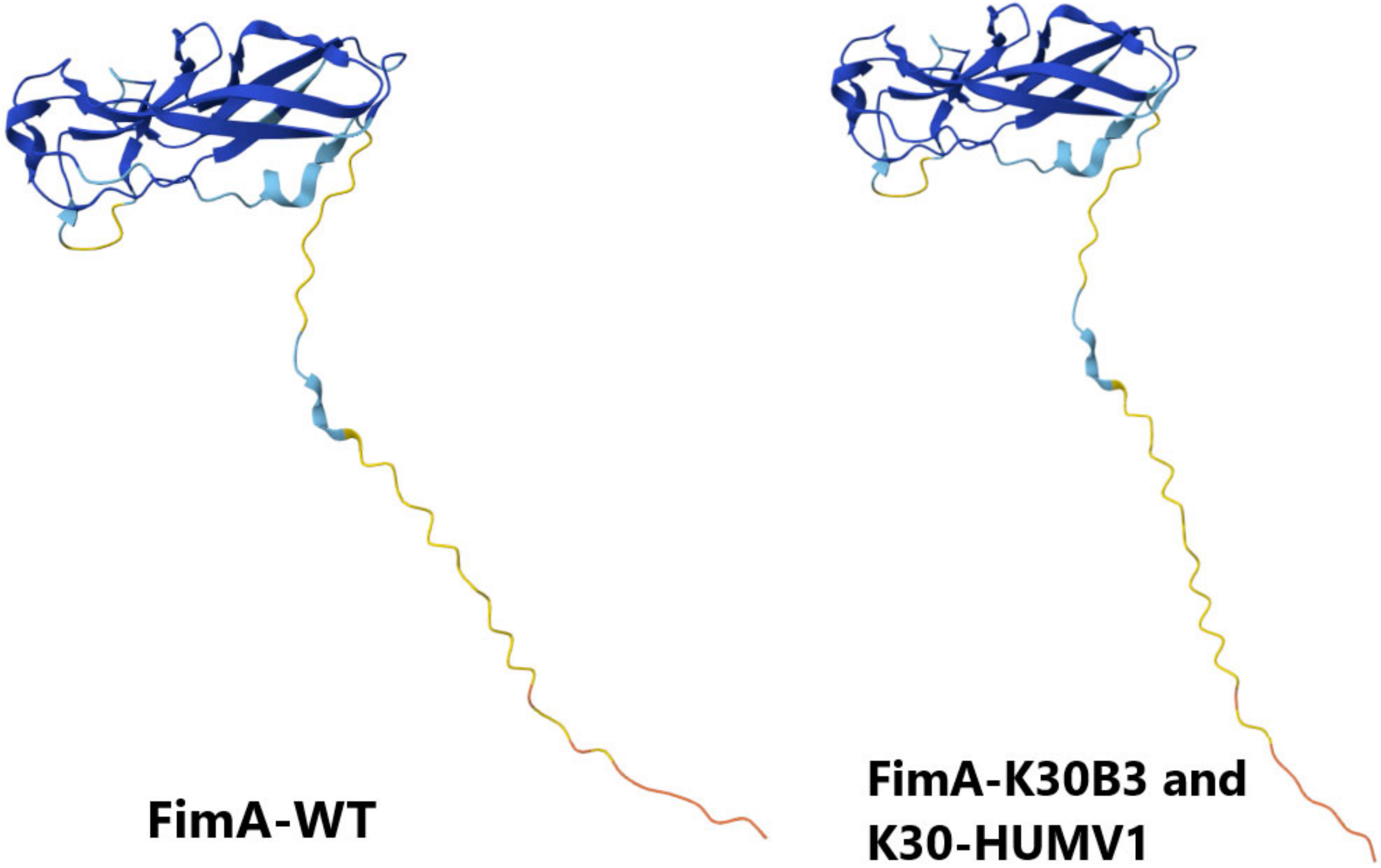

Supplementary Material. Figure S6. Predicted protein structures of wild-type and truncated FimA. Structural models were generated using AlphaFold v3.0.1 [30], the wild-type FimA protein of 182 amino acids was compared with the FimA protein variant from isolate K30B3 and K30-HUMV1. FimA protein from isolates K30B3 and K30-HUMV1 exhibited only three amino acid mutations (A19T, S74A, T151P). FimA-WT and the variants FimA-K30B3 and K30-HUMV1 display an almost identical structure without evident conformational differences. Models are colored by pLDDT confidence scores: blue (very high confidence), cyan–green (high to intermediate), yellow–orange (low), and red (very low confidence) in residue positioning.

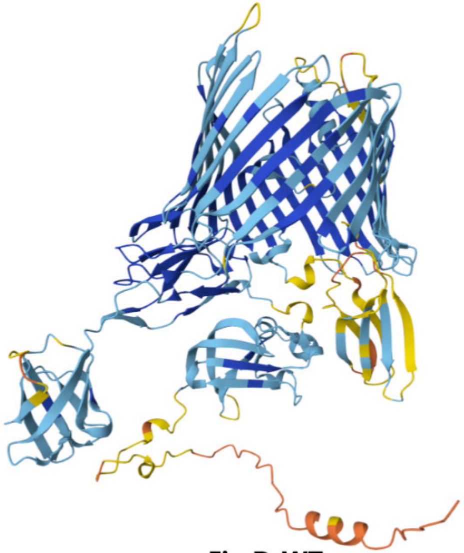

FimD-WT

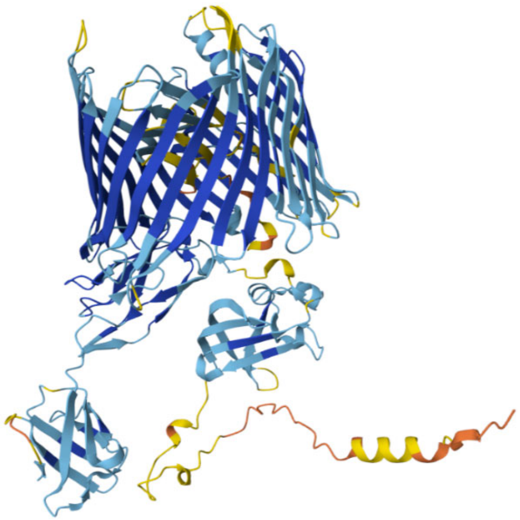

FimD-K30B1 and K30B3

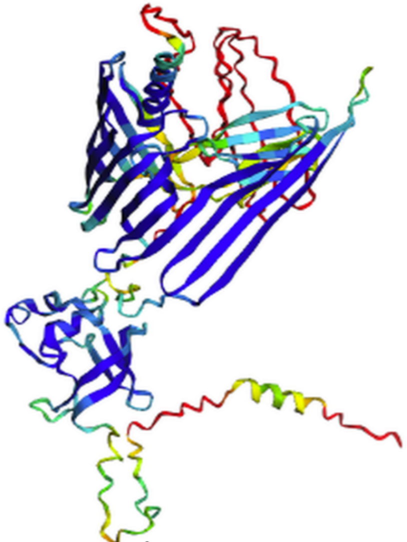

FimD-K30B2

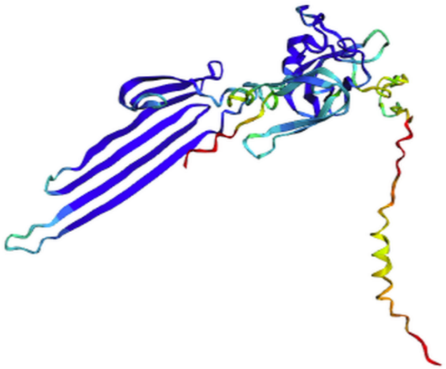

FimD-K30-HUMV1

Supplementary Material. Figure S7. Predicted protein structures of wild-type and truncated FimD. Structural models of WT FimD and the variant from K30B1 and K30B3 were generated using AlphaFold v3.0.1 [30], while the variants from K30B2 and K30-HUMV1 were generated using ColabFold v1.5.5 [31] since their structures were not represented in the AlphaFold database. The wild-type FimD protein of 828 amino acids was compared first with the FimD protein variant from isolate K30B1 and K30B3, which presented only two amino acid mutations (E48K, D436N), which displayed an identical structure. In contrast, isolates K30B2 and K30-HUMV1 exhibited truncated proteins of 577 and 329 amino acids, respectively, showing different alterations. These alterations suggest potential structural variability among these K30 variants that could influence FimD stability or function. Models are colored by pLDDT confidence scores: blue (very high confidence), cyan–green (high to intermediate), yellow–orange (low), and red (very low confidence) in residue positioning.
